# Different noses for different mice and men

**DOI:** 10.1186/1741-7007-10-75

**Published:** 2012-08-21

**Authors:** Andreas Keller

**Affiliations:** 1 The Rockefeller University, 1230 York Avenue, Box 63, New York, NY 10065, USA

## Abstract

Chemosensory receptor genes encode G protein-coupled receptors with which animals sense their chemical environment. The large number of chemosensory receptor genes in the genome and their extreme genetic variability pose unusual challenges for understanding their evolution and function. Two articles in *BMC Genomics *explore the genetic variation of chemosensory receptor gene repertoires in humans and mice and provide unparalleled insight into the causes and consequences of this variability.

See research articles http://www.biomedcentral.com/1471-2164/13/414 and http://www.biomedcentral.com/1471-2164/13/415

## Commentary

Humans sense odors with olfactory sensory neurons in the olfactory epithelium. Each olfactory sensory neuron expresses one G protein-coupled receptor from the large odorant receptor (OR) gene family. Different ORs have different ligand specificity and each odor therefore activates a different combination of sensory neurons. Many other mammals, including mice, have an additional olfactory organ, the vomeronasal organ. Instead of ORs, the sensory neurons in the vomeronasal organ express one gene from a repertoire of vomeronasal receptor (VR) genes. The VR gene repertoire consists of three families of G protein-coupled receptors: V1Rs, V2Rs, and formyl-peptide receptors (FPRs). The vomeronasal organ and the VRs play an important role in the detection of pheromones. Two articles in *BMC Genomics *present comprehensive characterizations of the entire human OR and mouse VR repertoire.

Wynn *et al*. [1] interrogated the rodent VR gene repertoire using massively parallel sequencing to sequence VRs in 17 inbred strains of mice. Thirteen were common lab strains that are mainly derived from *Mus musculus domesticus*. The other four were wild-derived strains of *Mus musculus *subspecies and a different mouse species, *Mus spretus*. The authors' analysis led to the identification of over 6,000 non-synonymous single nucleotide polymorphisms (SNPs) in 366 VR genes. Mouse VR genes are 2.3 times as variable as other mouse genes. Olender *et al*. [2] investigated the human OR repertoire by data-mining the 1000 Genomes Project and report similar results. The human OR genes are 2.5 times more variable than other genes in the human genome. The authors of this article identify almost 6,000 genetic variations, including SNPs, small indels, and structural variations, in 413 OR genes.

## Causes of genetic variability in chemosensory receptor genes

**Figure 1:**
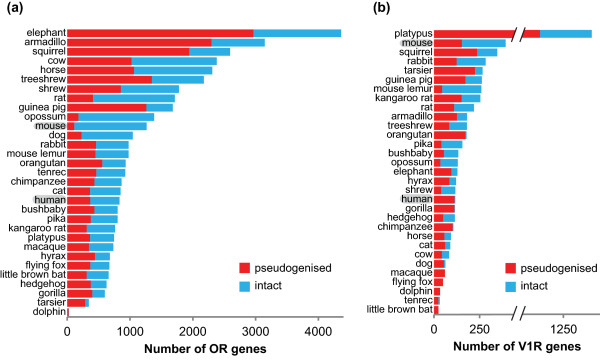
**Extreme variability in mammalian chemosensory receptor gene families**. **(a,b) **The OR gene family (a) and V1R gene families (b) of 31 mammalian species differ vastly both in the number of genes and in the percentage of intact genes. Much of this variability is caused by neutral genomic drift. However, there are also signs of selective processes. Note, for example, the very small percentage of intact V1R genes in humans and other primates. The articles by Olender *et al*. [2] and Wynn *et al*. [1] suggest that the clear division into intact and pseudogenized genes is appropriate only for single individuals. If the entire species is considered, many of the genes are found in both an intact and a pseudogenised version. Data in (a) from [4], in (b) from [5].

There are likely to be several evolutionary processes driving the high variability of chemosensory receptor genes, including a substantial contribution from neutral genomic drift, the process of random gene duplication, deletion, or inactivation [3]. Receptor genes duplicate at random and the duplicated gene, unless it confers an adaptive advantage, then mutates at random. Usually the duplicated gene will mutate into a non-functional variant, a so-called pseudogene, but sometimes it will mutate into a functional receptor gene with different ligand specificity than the receptor from which it arose. Genomic drift explains why chemosensory receptor gene families are extremely large, extremely variable, and contain a high percentage of pseudogenes [4,5] (Figure 1).

It has been a challenge to identify directed evolutionary processes that shaped the chemosensory receptor gene repertoires in the background of the variability resulting from undirected effects of genomic drift . The datasets analyzed by Olender *et al*. [2] and Wynn *et al*. [1] are large enough to detect signatures of diverse selective processes. Olender *et al*. [2] demonstrate that 57 human ORs show evidence of strong purifying selection - that is, selection against non-functional variants - indicating that it is an adaptive advantage to have a functional variant of these ORs. A smaller group of ORs shows patterns of genetic variability suggestive of balancing selection, a process by which multiple alleles of the same gene are actively maintained in the human gene pool. Although previous studies reported positive selection acting on specific ORs, in this study no clear evidence for positive selection was found.

Similar results were obtained by Wynn *et al*. [1] for the mouse VR genes. The large increase in VR variability in domesticated mouse strains could simply be a consequence of the removal of selective constraints on VR genes during domestication. However, Wynn *et al*. [1] provide evidence that this is not the case. If VR genes were free from selective constraints, the distribution of variation would be uniform; however, this is not the case. Instead there are complex patterns of non-random sequence variation that indicate that mouse VRs, like human ORs, are under diverse selective pressures.

## Consequences of genetic variability in chemosensory receptor genes

Genomic drift with purifying selection that acts only on a subset of the genes and a combination of balancing and perhaps also positive selection result in genetically highly diverse chemosensory receptors. What are the consequences of this genetic variability? In humans, genetic diversity will result in perceptual diversity. Each individual perceives olfactory stimuli with their personal set of ORs. Olender *et al*. [2] identified 244 ORs for which both an intact and a pseudogenized version are found in the population. In two randomly selected individuals one third of OR alleles will be functional in one individual but non-functional in the other individual. These huge differences in the sensory apparatus are very likely to have dramatic influences on how odorous stimuli are perceived and it has already been shown that, in some cases, genetic variability in ORs influences how strong and pleasant the ligands of the ORs are perceived to be [6].

Similar to the findings in human ORs, Wynn *et al*. [1] found that, for a large percentage of mouse VR genes, both an intact version and a version that is truncated or carries a frame shift mutation are present. The repertoire of functional VRs with which different mouse strains perceive stimuli therefore varies significantly. Because mouse VRs sense chemical signals that are used in social communication and often have pheromonal activity [7], this variability in VR genes may result in variability in pheromone-mediated behaviors. Differences in such behaviors have been observed between the mouse strains that were analyzed by Wynn *et al*. [1] and it is tempting to speculate that the genetic differences in the VRs contribute to these behavioral differences. However, to fully understand the relationship between genetic VR variation and behavioral variability, the ligands that bind to the VRs have to be identified.

Although for most VRs the ligands are unknown, groups of V2Rs that respond preferentially to conspecific odor cues, odors from other rodent species and subspecies, or predator odors have recently been identified [8]. Interestingly, Wynn *et al*. [1] found more genetic variability in V2Rs that are tuned to olfactory signals from other mouse subspecies than in receptors tuned to the smell of predators. Based on this finding the authors speculate that genetic variability in V2Rs could be a driving force of speciation by mediating a behavioral barrier that contributes to reproductive isolation between subspecies. A similar process has been described in moths, where genetic variability in pheromone receptors contributes to speciation [9]. Rather than being inconsequential noise in highly redundant genes, chemosensory receptor gene variability may in some cases be driving sympatric speciation.

We are only just beginning to understand the causes and consequences of the unusual genetic and functional variability of large chemosensory receptor gene repertoires in different species. The comprehensive data on genetic variability in the human OR and mouse VR repertoire presented in the articles by Olender *et al*. [2] and Wynn *et al*. [1] will be invaluable in tackling these questions.
